# Hospital-Wide Multidisciplinary, Multimodal Intervention Programme to Reduce Central Venous Catheter-Associated Bloodstream Infection

**DOI:** 10.1371/journal.pone.0093898

**Published:** 2014-04-08

**Authors:** Walter Zingg, Vanessa Cartier, Cigdem Inan, Sylvie Touveneau, Michel Theriault, Angèle Gayet-Ageron, François Clergue, Didier Pittet, Bernhard Walder

**Affiliations:** 1 Infection Control Programme, University of Geneva Hospitals, Geneva, Switzerland; 2 Division of Anaesthesiology, University of Geneva Hospitals, Geneva, Switzerland; 3 Nursing Department, University of Geneva Hospitals, Geneva, Switzerland; 4 WHO Collaborating Centre on Patient Safety, University of Geneva Hospitals and Faculty of Medicine, Geneva, Switzerland; University of Calgary, Canada

## Abstract

Central line-associated bloodstream infection (CLABSI) is the major complication of central venous catheters (CVC). The aim of the study was to test the effectiveness of a hospital-wide strategy on CLABSI reduction. Between 2008 and 2011, all CVCs were observed individually and hospital-wide at a large university-affiliated, tertiary care hospital. CVC insertion training started from the 3^rd^ quarter and a total of 146 physicians employed or newly entering the hospital were trained in simulator workshops. CVC care started from quarter 7 and a total of 1274 nurses were trained by their supervisors using a web-based, modular, e-learning programme. The study included 3952 patients with 6353 CVCs accumulating 61,366 catheter-days. Hospital-wide, 106 patients had 114 CLABSIs with a cumulative incidence of 1.79 infections per 100 catheters. We observed a significant quarterly reduction of the incidence density (incidence rate ratios [95% confidence interval]: 0.92 [0.88–0.96]; *P*<0.001) after adjusting for multiple confounders. The incidence densities (n/1000 catheter-days) in the first and last study year were 2.3/1000 and 0.7/1000 hospital-wide, 1.7/1000 and 0.4/1000 in the intensive care units, and 2.7/1000 and 0.9/1000 in non-intensive care settings, respectively. Median time-to-infection was 15 days (Interquartile range, 8-22). Our findings suggest that clinically relevant reduction of hospital-wide CLABSI was reached with a comprehensive, multidisciplinary and multimodal quality improvement programme including aspects of behavioural change and key principles of good implementation practice. This is one of the first multimodal, multidisciplinary, hospital-wide training strategies successfully reducing CLABSI.

## Introduction

Central vascular lines are indispensable in hospital care, but the major potential complication of their use is central line-associated bloodstream infection (CLABSI) [1], [2]. Risk factors for infection include catheter-dwell time, access site, multi-lumen catheters, the patient's underlying conditions, as well as catheter care practices [3]. Most studies and interventions for the prevention of central venous catheter (CVC) infection are performed in intensive care units (ICU). However, hospital-wide surveillance activities in our institution revealed that although most CVCs are inserted in the ICU, two-thirds of CVC days accumulate in non-ICU wards [4]. Indeed, the incidence of CLABSI is reported to be even higher in some non-ICU settings in the rare studies where surveillance included all hospital wards [4]–[6], thus stressing the need for hospital-wide surveillance and prevention activities. Many studies conducted in the ICU have shown that bundle strategies or multimodal intervention programmes reduced CLABSI rates by emphasizing best practice for catheter insertion and care [7]–[14]. The current study evaluated the effectiveness of a hospital-wide, multimodal, prevention strategy on the reduction of CLABSI reduction.

## Methods

### Setting and Study Design

This prospective study was conducted between 2008 and 2011 at the University of Geneva Hospitals, Geneva, Switzerland, a 1908-bed primary and tertiary care centre. In 2011, there were 48,112 admissions accounting for 671,709 hospital-days. All adult inpatients with a CVC were eligible for study inclusion. The policies regarding management of suspected CLABSI and the method for obtaining blood cultures did not change during the study period. Outcomes were stratified by department (ICU, surgery, internal medicine).

### Ethics Statement

The study was approved by the institutional review board of the University of Geneva Hospitals, Geneva, Switzerland (protocol number 07023). The institutional review board waived the need for written informed consent from the participants.

### Intervention

In 2007, existing protocols related to CVC insertion and care were reviewed and updated by an interdisciplinary study group, which included members from anaesthesiology, infection control, and the nursing department. A detailed insertion checklist was defined by the study group based on evidence in the literature and by repeated practice testing in daily routine. The complete insertion procedure from patient preparation until dressing application was filmed for training purposes. For catheter care, a modular e-learning programme was developed, including assistance with CVC insertion, infusate preparation, CVC manipulation, dressing change, CVC removal, and clinical surveillance and documentation (www.carepractice.net). All modules featured detailed procedure sequences and were animated by short movies, icons, and keywords.

From 2008, dedicated CVC insertion carts and complete single-use kits were introduced hospital-wide. The carts were stocked with all equipment for catheter insertion and served as a movable working surface. Insertion kits were designed to follow the procedure sequence of aseptic skin preparation and CVC insertion; the first upper level contained the material for skin preparation, and the second level included all the necessary equipment for CVC insertion. Neither antiseptic- or antibiotic-impregnated catheters nor chlorhexidine-impregnated dressings were used at any time during the study. Alcohol-based chlorhexidine for skin antisepsis has been an established procedure in the entire hospital before the study.

After a baseline of 6 months, physicians working in the operating theatre and intensive care were trained during 4-hour workshops at the hospital simulator training centre (simulHUG: http://simulationmedicale.hug-ge.ch/). The workshop was divided into 3 sequences: 1) lecturing on CVC insertion and CLABSI prevention; 2) filming of participants inserting a CVC; and 3) giving feedback based on best practice recommendations. Between October 2009 and March 2010, all nurses in the medical and surgical wards were trained by their supervisors using the “carepractice” e-learning programme. Supervisors were familiarized with the e-learning programme in focus groups where they also had to prove their capacity to organize appropriate training sessions. All new medical and nursing staff were trained using the tools as described above.

### Outcome measures

Detailed CVC surveillance was conducted by trained infection control nurses and included CVC type, insertion site, and dwell time. All relevant information was recorded in a surveillance case report form for each patient. Bloodstream infection defined as bacteraemia (or fungaemia) in the presence of a CVC with no other apparent source was the primary outcome. For coagulase-negative staphylococci (CoNS), two positive blood cultures or a complete course of antibiotic therapy adjusted for susceptibility testing were required [2]. There was no change in definitions during the study period. All patients were monitored for CLABSI until 48 h following CVC removal. All-cause mortality at day 28 after CVC removal was the secondary outcome.

### Sample size estimation

On the basis of the baseline incidence of 4.2 CLABSI episodes per 1000 catheter-days (15 CLABSI episodes per 426 CVCs) observed during the pilot study [4], we calculated that a sample size of at least 1409 CVCs accumulating 12,110 catheter-days would be necessary in both the baseline and the intervention period to test the assumption of a 50% reduction in CLABSI incidence taking an alpha error at 5% and a study power of 80%.

### Statistical analysis

Categorical variables were compared using the chi-square test; continuous variables were summarized as medians and compared using the Wilcoxon rank sum test. CLABSI incidence rates were studied across time by using a mixed-effects Poisson regression analysis and reported as incidence rate ratios (IRR). As patients could be hospitalized several times and receive multiple CVCs, the model took into consideration a random effect at the individual patient level. All risk factors were assessed first by univariable analysis and variables of clinical interest were included in the multivariable analysis. Potential confounders for adjusted time trend analysis were gender, patient age, Charlson co-morbidity index [15], length of hospital stay, length of ICU stay, emergency admission, operator, CVC type, insertion site, and catheter dwell time.

All-cause mortality was assessed up to day 28 after removal of the last CVC using logistic regression analysis adjusted for gender, patient age, Charlson co-morbidity index, emergency admission, ICU stay, CLABSI, and number of CVCs during hospital stay. Results are reported as odds ratio (OR) and their 95% confidence interval (95% CI). Two-sided *P*-values of less than 0.05 were considered to indicate statistical significance. All statistical analyses were conducted using Stata software, version 12.0 (StataCorp).

## Results

**Figure 1:**
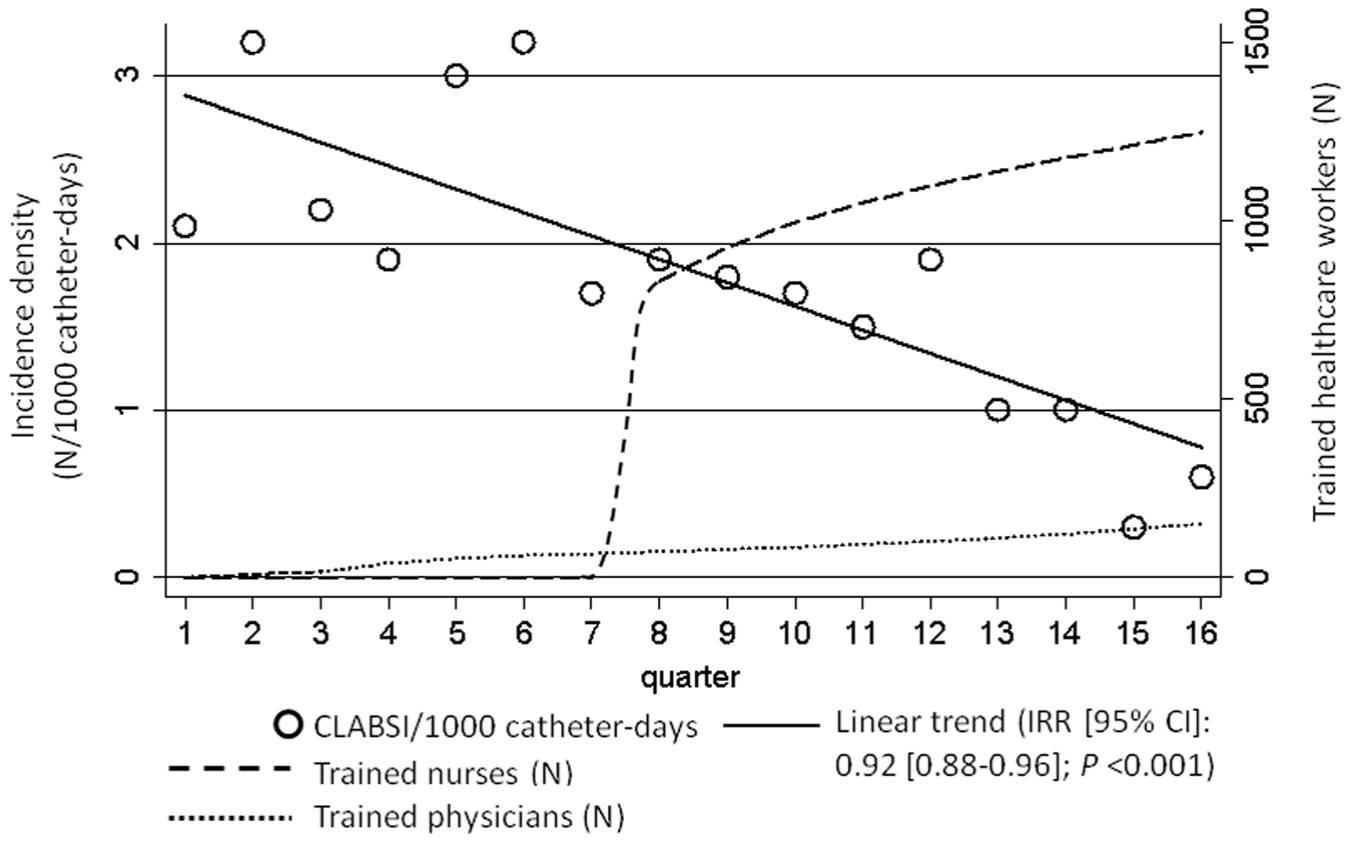
Central Line-Associated Bloodstream Infections and Number of Trained Healthcare Workers, Hospital-wide Prevention Programme, University of Geneva Hospitals, 2008–2011. CLABSI: central line-associated bloodstream infection IRR: incidence rate ratio 95% CI: 95% confidence interval.

Between June 2008 and November 2011, 146 physicians were trained in 36 simulator-based workshops (Figure 1). Between October 2009 and March 2010, 980 nurses were trained by the “carepractice” tool, and an additional 294 newly-employed nurses were trained (Figure 1).

A total of 189,643 patients were admitted to our institution during the study period. Among these, 3952 (2.1%) received at least one CVC. Table 1 summarizes characteristics of patients included in the study and the presence or absence of CLABSI: there was no significant difference in patient characteristics, such as age, gender, and the Charlson co-morbidity index, between patients with and without CLABSI. Hospital and ICU lengths of stay were significantly longer among patients with CLABSI (Table 1).

**Table 1:** Characteristics of Patients With and Without Central Line-Associated Bloodstream Infection, University of Geneva Hospitals, 2008–2011.

	All patients#	Patients without CLABSI	Patients with CLABSI	*P* value
Total number of patients, N	3952	3846	106	NA
Total number of hospitalizations, N	4452	4343	109	NA
Age, median (IQR)	64 (50–75)	64 (50–75)	60.5 (47–74)	.101
Male gender, N (%)	2337 (59.1)	2279 (59.3)	58 (54.7)	.348
Charlson co-morbidity index, median (IQR)	4 (2–6)	4 (2–6)	4 (2–7)	.188
*Length of hospital stay, median (IQR)	23 (14–42)	23 (13–41)	57 (31–83)	<.001
*ICU stay, N (%)	2889 (64.9)	2843 (65.5)	46 (42.2)	<.001
*ICU stay, median (IQR)	2 (0–4)	2 (0–4)	2 (0–3)	.002
*Emergency admission, N (%)	843 (18.9)	824 (19.0)	19 (17.4)	.685
*Death at day 28, N (%)	701 (17.7)	683 (17.8)	18 (17.0)	.836

CLABSI: central line-associated bloodstream infection.

NA: not applicable.

IQR: interquartile range.

ICU: intensive care unit.

# A total of 189,643 patients were admitted during the study period.

*As per hospitalization (n = 4,452).

A total of 6352 CVCs were placed in 3952 patients during 4452 hospitalizations. CVC characteristics with unadjusted yearly time trends are shown in Table 2. Two- (38.2%) and 3- (21.4%) lumen catheters were most commonly used. All multi-lumen catheters accumulated to 4207 (66.2%). Total accumulated dwell-time was 61,366 catheter-days with 23,286 (37.9%) within the ICU and 38,080 (62.1%) outside the ICU. The CVC utilization ratios in the ICU and outside the ICU were 56.6%, and 1.5%, respectively. The majority of patients were not transferred from one department to another with their CVC in place and thus, 84.3% of CVCs were exposed to one department only.

**Table 2:** Catheter Characteristics with Yearly Unadjusted Time Trends: Hospital-wide Prevention Programme for Central Line-Associated Bloodstream Infections, University of Geneva Hospitals, 2008–2011.

	All CVCs	Time trend, IRR (95% CI)	*P* value
Total number of catheters, N	6352	NA	NA
Dwell time, median (IQR)	6 (3–12)	1.05 (1.04–1.06)	<.001
Jugular position, N (%)	4272 (67.3)	1.07 (1.04–1.10)	<.001
Subclavian position, N (%)	1319 (20.8)	0.86 (0.81–0.90)	<.001
Femoral position, N (%)	761 (12.0)	0.89 (0.84–0.95)	.001
Multilumen catheters, N (%)	4207 (66.2)	1.06 (1.04–1.10)	<.001
Placed in the operating theatre, N (%)	2913 (45.9)	1.00 (0.97–1.04)	.929
Placed in the ICU, N (%)	2647 (41.7)	0.97 (0.94–1.01)	.116
Placed in a non-ICU ward, N (%)	786 (12.4)	1.09 (1.02–1.16)	.007
Dwell-time^3^ in ICU, median (IQR)	4 (2–7)	1.06 (1.05–1.07)	<.001
Dwell-time^3^ in non-ICU settings, median (IQR)	9 (4–17)	1.06 (1.05–1.07)	<.001
Dwell-time^3^ in medical wards^1^, median (IQR)	10 (5–20)	1.05 (1.04–1.06)	<.001
Dwell-time^3^ in surgical wards^2^, median (IQR)	8 (3–15)	1.06 (1.05–1.08)	<.001

CVC: central venous catheter.

ICU: intensive care unit.

IQR: interquartile range.

IRR: incidence rate ratio.

NA: not applicable.

95% CI: 95% confidence interval.

1 Medicine: internal medicine, neurology, rehabilitation.

2 Surgery: cardiovascular, thoracic and abdominal surgery, orthopaedics, neurosurgery, urology, ear-nose-throat, trauma surgery.

3 Dwell-time: catheter-days.

One hundred and six patients had a total of 114 CLABSI over the entire study period with significant quarterly reductions after adjustment for major confounders (Figure 1; Table 3). CLABSI incidence densities (episodes/1000 catheter-days) in the first and last study year were 2.3/1000 and 0.7/1000 hospital-wide, 1.7/1000 and 0.4/1000 in the ICU, and 2.7/1000 and 0.9/1000 in non-ICU settings, respectively. Median time-to-infection was 15 days (IQR, 8–22). Although most CLABSI were identified among CVCs in place for 12 days or longer (64/114), a significant association was identified only for catheters in place for 7–12 days. This was due to the fact that this group had the highest incidence density compared to the 2^nd^ and 4^th^ quartiles (2.3/1000 vs. 1.9/1000 and 1.8/1000 catheter-days, respectively) for which a trend was calculated (Table 3). No association with CLABSI was identified for the femoral position (Table 3), and their number significantly decreased over time (Table 2). A total of 130 pathogens were isolated from 114 CLABSI episodes and their distribution is summarized in Table 4. Fourteen percent (16/114) was the proportion of polymicrobial infections. All-cause mortality up to 28 days after removal of the last CVC was associated with age, the Charlson co-morbidity index, emergency admission, ICU stay, and a higher number of CVCs during hospital stay (Table 5). No significant positive or negative time trends for all-cause mortality were identified across the study period. The rates of yearly blood culture samples per 1000 patient-days in 2008, 2009, 2010, and 2011 were 38.6, 39.5, 37.9, and 42.9, respectively.

**Table 3:** Factors Associated with Central Line-Associated Bloodstream Infections: Hospital-wide Prevention Programme for Central Venous Catheter-Associated Bloodstream Infections, University of Geneva Hospitals, 2008–2011.

	Univariable model			Multivariable model		
	IRR	95% CI	*P*-value	IRR	95% CI	*P* value
Quarter^1^	0.92	0.88–0.96	<0.001	0.92	0.88–0.96	<.001
Age^2^	0.99	0.99–1.01	0.772	0.99	0.98–1.01	.301
Gender^3^	0.91	0.63–1.33	0.637	0.92	0.63–1.35	.658
Charlson comorbidity index^4^	1.04	0.989–1.11	0.143	1.07	0.99–1.14	.065
ICU stay^5^	0.82	0.56–1.20	0.298	1.21	0.71–2.07	.475
Multilumen catheters^6^	1.44	0.87–2.40	0.159	1.47	0.87–2.47	.146
Femoral position^7^	1.26	0.73–2.19	0.407	1.22	0.69–2.14	.492
Dwell-time (4–6 days)^8^	2.78	0.80–9.69	0.108	3.12	0.89–10.95	.075
Dwell-time (7–12 days)^8^	3.53	1.08–11.52	0.037	3.81	1.15–12.63	.029
Dwell-time (>12 days)^8^	2.97	0.93–9.46	0.066	3.03	0.91–10.09	.070
Placed in the ICU	0.65	0.42–1.01	0.056	0.51	0.29–0.90	.020

ICU: intensive care unit

IRR: incidence rate ratio.

95% CI: 95% confidence interval.

1 Quarter: modelled as per additional quarter.

2 Age: modelled as per additional year of age.

3 Gender: modelled as male vs. female.

4 Charlson score: modelled as per score-point increase.

5 ICU stay: hospitalization in the intensive care unit; modelled as yes vs. no.

6 Multilumen catheters: any catheter with more than 1 lumen; modelled as yes/no.

7 Femoral position: any catheter inserted at the femoral site; modelled as yes/no.

8 Dwell-time (quartiles): 2^nd^ (4–6 days), 3^rd^ (7–12 days), and 4^th^ (>12 days) quartile as compared with the first quartile (1–3 days).

**Table 4:** Distribution of Pathogens: Hospital-wide Prevention Programme for Central Line-Associated Bloodstream Infection, University of Geneva Hospitals, 2008–2011.

Pathogen	N	%
Coagulase-negative staphylococci	41	31.5
Methicillin-resistant *Staphylococcus aureus*	17	13.1
Methicillin-susceptible *Staphylococcus aureus*	16	12.3
*Enterococcus* spp	12	9.2
*Klebsiella* spp	9	6.9
*Pseudomonas* spp	9	6.9
*Candida albicans*	8	6.2
*Escherichia coli*	4	3.1
*Proteus* spp	3	2.3
*Acinetobacter* spp	2	1.5
*Serratia* spp	2	1.5
Others	7	5.4
Total	130	100.0

**Table 5:** 28-Day All-Cause Mortality: Hospital-wide Prevention Programme for Central Line-Associated Bloodstream Infection, University of Geneva Hospitals, 2008–2011.

	Univariable model			Multivariable model		
	OR	95% CI	*P* value	OR	95% CI	*P* value
Quarter^1^	1.00	0.99–1.02	.956	1.00	0.98–1.01	.652
Age^2^	1.02	1.02–1.03	<.001	1.01	1.01–1.02	<.001
Gender^3^	1.15	1.00–1.33	.053	1.09	0.95–1.24	.233
Charlson index^4^	1.13	1.11–1.15	<.001	1.08	1.05–1.11	<.001
Emergency admission^5^	1.44	1.23–1.67	<.001	1.34	1.15–1.55	<.001
ICU stay^6^	3.73	2.97–4.70	<.001	3.19	2.53–4.02	<.001
CLABSI^7^	0.75	0.45–1.26	.281	0.66	0.40–1.07	.091
CVC count^8^	1.20	1.16–1.25	<.001	1.14	1.09–1.19	<.001

CI: confidence interval.

CLABSI: central line-associated bloodstream infection.

CVC: central venous catheter.

ICU: intensive care unit.

OR: odds ratio.

95% CI: 95% confidence interval.

1 Quarter: modelled as per additional quarter.

2 Age: modelled as per additional year of age.

3 Gender: modelled as male vs. female.

4 Charlson index: modelled as per score-point increase.

5 Emergency admission: modelled as yes/no.

6 ICU stay: hospitalization in the intensive care unit at any time; modelled as yes/no.

7 Central line-associated bloodstream infection at any time during hospitalization; modelled as yes/no.

8 Number of CVCs during hospitalization; modelled as per additional catheter.

## Discussion

In a setting with low baseline rates, the incidence of CLABSI was reduced by a hospital-wide, best practice-oriented prevention programme, including the ICU [1], [16]. This is one of the first multidisciplinary, multimodal hospital-wide training strategies to show significant CLABSI reduction in a high-income country.

The incidence density of CLABSI in our ICU is the same (0.4/1000 catheter days) as the very low incidence reported in the literature by Timsit and colleagues in the intervention arm of a large randomized, controlled study demonstrating the benefit of chlorhexidine-impregnated dressings [17], and also lower than that achieved by Pronovost and colleagues (1.1/1000 catheter-days) using a bundle strategy targeting catheter insertion [18]. The only published hospital-wide (ICU and non-ICU together) CLABSI prevention study is from Thailand [19]. The study addressed hand hygiene by posters and lectures, improved catheter insertion by using full barrier precautions, avoided femoral catheter use, and applied a system to remove catheters as soon as possible. It introduced also a chlorhexidine-containing disinfectant for skin antisepsis. The baseline incidence density of 14 episodes per 1000 catheter-days was very high, even for a country with limited resources [20]. Thus, the two settings are not comparable. A quasi-experimental study in the non-ICU wards of 11 Spanish hospitals resulted in a significant reduction of the overall incidence of CLABSI and BSI related to peripheral lines (0.19/1000 patient-days vs. 0.15/1000 patient-days) [21]. The strategy followed the bundle promoted by Pronovost and colleagues [9], adding aspects of catheter-care and specific recommendations for peripheral lines. More than 2000 healthcare workers were trained at the 11 sites. Another recent study combining the same bundle with the “Comprehensive Unit-based Safety Programme” reported significant reductions of CLABSI-rates from 4.52/1000 catheter-days to 0.25/1000 catheter-days in 14 non-ICU wards in Hawaii [22].

Our intervention programme addressed catheter care by taking into consideration aspects of behaviour change and key principles of good implementation practice [23], [24]. Programme implementation included a number of factors shown to be effective in the successful implementation of infection control strategies, such as fostering multidisciplinary collaboration [7], [25]–[27], involving different professional categories [28], encouraging leadership [29], [30], and encouraging the hospital administration to play an active role [30]. Physicians participating in the study team and conducting the simulation training were also part of the clinical team and acted as role models in daily practice [31], [32]. The CVC insertion cart offering all necessary material for catheter insertion at a single place and the new insertion set designed to logically follow the insertion sequence also contributed to success [33], [34].

Simulation-based training of catheter insertion allowed the adoption of evidence-based techniques in a stress-free environment, similar to other reports where it effectively improved knowledge and insertion technique [35]–[40]. Reduction of CLABSI by 84% (from 3.2 to 0.5/1000 catheter-days; *P*<.001) and 71% (from 3.5 to 1.0/1000 catheter-days; *P*<.001) has been described in the ICU setting, but not in other wards [35], [38]. The decision to use an e-learning module for nurse training was pragmatic given the large number of professionals to be trained hospital-wide. We assume that the “train-the-trainer” model helped professionals to identify with the teacher and allowed a “buy-in” of the strategy on the wards as this has been successful also in other studies and settings [41], [42]. The e-learning tool itself (www.carepractice.net) comprehensively addressed every detail of catheter care. The design and structuring prevented the tool from looking overloaded and simplified navigation through the modules. Informal feedback about the tool from healthcare professionals was highly favourable.

Similar to a recent study among haemodialysis patients [43], but in contrast to a number of older studies [44]–[46], we did not find an association between CVCs placed in the femoral vein and CLABSI. However, we found a significant association for the 2^nd^ quartile of catheter dwell-time (7 to 12 days). CVCs with longer dwell-times accumulated more catheter-days and although more CLABSIs were identified, the incidence density was lower. CVCs with lower dwell-times accumulated less catheter-days, but also had less CLABSIs, which resulted in a lower incidence density as well. Our data suggest that the highest risk is for catheters in place for 7 to 12 days and support the idea of leaving CVCs in place until they are no longer required.

Crude mortality in our study (17.7%) was higher than in the hospital-wide Thai study (11–12%) [19], but lower than in a recent report among non-ICU patients (23%) [47]. However, we could not identify attributable mortality for CLABSI or observe any association with crude mortality. Similar to the study by Apisarnthanarak and colleagues, mortality did not change over time (Table 5). Our findings challenge older reports of very high crude and attributable mortality due to CLABSI in ICU settings [48] [49].

Our prevention programme can and should be applied by other hospitals with quality improvement interest; in particular, because the core element of our programme “carepractice.net” is freely accessible. We believe that the concept of combining practical training for a smaller specialized group with e-learning for a larger group can be adapted to other areas in infection control, such as the prevention of catheter-associated urinary tract infection or surgical site infection.

### Study limitations

Our study has limitations. First, the study was non-controlled and observational, and thus, a regression-to-the-mean-effect may have occurred. However, the baseline incidence density was not unusually high (2.3/1000 catheter-days) for a tertiary-care hospital in a high-income country and the final analysis was adjusted for a large number of potential confounders, such as gender, age, comorbidities, catheter type and site placement, professionals placing the catheter, and variations of dwell-time. Although confounders could be better controlled in a randomized controlled trial, a study addressing behaviour change of entire groups of professionals cannot be conducted in a single centre using such a design. Second, generalisability is limited based on data coming from a single centre. However, the University of Geneva Hospitals is a large institution providing both primary and tertiary care with a broad patient population case- mix. Third, unfortunately, there were no consistent process indicators; this was mainly due to the high workload for catheter surveillance. Although process measures would have added strength to the findings of the study, the results of the multivariable analysis adjusting for a number of case-mix variables and the fact that no technology (coated catheter, impregnated sponges, other skin disinfectants, other insertion sets) was introduced during the four-year study period makes the intervention very likely to be responsible for a large proportion of the positive effect.

### Conclusion

Our findings suggest that clinically relevant reduction of hospital-wide CLABSI was reached with a comprehensive, multidisciplinary and multimodal quality improvement programme including aspects of behavioural change in a practical manner and key principles of good implementation practice. The content of our training programmes was comprehensive and addressed all single steps in the procedure of CVC insertion and catheter care. In this sense, our strategy is more in-depth than some promoted bundles [9], [12], [18]. Where comparable, our infection rates are among the lowest published in the literature and obtained without the use of antiseptic- or antibiotic-impregnated catheters dressings. Our results support the idea that complex medical procedures should be addressed comprehensively rather than by simplified approaches. More efforts should be invested in understanding how a prevention programme is adopted and implemented in daily practice [24].
